# Psychometric Properties of the Persian Version of Death Depression Scale-Revised in Iranian Patients with Acute Myocardial Infarction

**Published:** 2017-07

**Authors:** Hamid Sharif Nia, Saeed Pahlevan Sharif, Rebecca H. Lehto, Kelly A. Allen, Amir Hossein Goudarzian, Ameneh Yaghoobzadeh, Mohammad Ali Soleimani

**Affiliations:** 1 School of Nursing and Midwifery Amol, Mazandaran University of Medical Sciences, Sari, Iran.; 2 Senior Lecturer, Taylor’s Business School, Taylor’s University Malaysia, Subang Jaya, Malaysia.; 3 Michigan State University College of Nursing, Michigan, USA.; 4 The Melbourne Graduate School of Education, The University of Melbourne, Melbourne, Australia.; 5 Nursing, Student Research Committee, Mazandaran University of Medical Sciences, Sari, Iran.; 6 Master in Geriatric Nursing, Tehran University of Medical Sciences, Tehran, Iran.; 7 Social Determinants of Health Research Center, Qazvin University of Medical Sciences, Qazvin, Iran.

**Keywords:** *Acute Myocardial Infarction*, *Death Depression Scale*, *Psychometric*, *Persian Version*

## Abstract

**Objective:** Limited research has examined the psychometric properties of death depression scales in Persian populations with cardiac disease despite the need for valid assessment tools for evaluating depressive symptoms in patients with life-limiting chronic conditions. The present study aimed at evaluating the reliability and validity of the Persian Version of Death Depression Scale - Revised (DDS-R) in Iranian patients who had recent acute myocardial infarction (AMI).

**Method:** This psychometric study was conducted with a convenience sample of 407 patients with AMI diagnosis who completed the Persian version of the DDS-R. The face, content, and construct validity of the scale were ascertained. Internal consistency, test–retest, and construct reliability (CR) were used to assess reliability of the Persian Version of DDS-R.

**Results:** Based on maximum likelihood exploratory factor analysis and consideration of conceptual meaning, a 4-factor solution was identified, explaining 75.89% of the total variance. Goodness-of-fit indices (GFI), Comparative Fit Index (CFI), Normed Fit Index (NFI), Incremental Fit Index (IFI), and Root Mean Square Error of Approximation (RMSEA) in the final DDS-R structure demonstrated the adequacy of the 4-domain structure. The internal consistency, construct reliability, and Intra-class Correlation Coefficients (ICC) were greater than .70.

**Conclusion:** The DDS-R was found to be a valid and reliable assessment tool for evaluating death depression symptoms in Iranian patients with AMI.

The awareness and acceptance of mortality is a ubiquitous human concern (1, 2). Perceptions, beliefs, and attitudes about death contribute to aversive affect and subsequent adaptation to life-threatening illness (3). Given the impact that grappling with the proximity of death has on psychological health, Templer and associates in 1990 introduced the concept of death depression to describe the sadness one may report in connection to thoughts concerning the death of themselves, the death of others, or death in general. Irrespective of whether death is imminent for an individual or not, most people experience aversive affect associated with thoughts about death at some point during their life (4, 5). Depressive perceptions associated with death have been reported across ages, cultures, and religions, making it a global concern for practitioners and medical professionals who interact closely with patients (6).

Humans are recognized to report anxiety and/or despair relating to the reality of death following a life threatening event (7). Cardiac events, in particular, such as acute myocardial infarction (AMI) are often accompanied by severe psychological sequelae (8). AMI, an occlusion of a major coronary artery with subsequent anoxia and cardiac muscle necrosis, (9) is now the second most common cardiovascular disease in both developed and developing countries worldwide (10). In Iran, about 3.6 million patients are affected (11).

Psychological sequelae are common following AMI, with depressive symptoms being the most prevalent (12, 13). Research has found that depression following AMI is associated with decreased adherence to post-event recommendations to reduce risk and manage health behaviors to avoid further negative outcomes of the cardiovascular disease (14, 15). Thus, depression may indirectly contribute to the progression of cardiovascular disease as well as impede general, physical, and psychological recovery (8, 16, 17). Importantly, depression is diagnosed in patients following AMI at rates 3 times higher than in the general population (18, 19). 

Aversive cognitions and emotions about death can be associated with depressive symptoms (20). The Death Depression Scale was developed to address the need for a valid tool to effectively measure the presence of depressive symptoms such as sadness and grief associated with perceptions about the reality of death (6, 21). Death Depression Scale-Revised (DDS-R), has strong psychometric properties with subsequent translations and testing among Arabic (5, 22) and Spanish (21) people. The empirical research has shown that this scale has adequate internal consistency, good discriminatory validity, and a meaningful factor structure (23). The DDS-R has been evaluated in conjunction with other validated depression measures demonstrating the evidence of construct validity (24).

Given the high prevalence of depression in patients who have experienced an AMI, it is essential that depressive cognitions and affect associated with thoughts about death and dying is available. Given the life-threatening nature of cardiac infarction, fears associated with mortality are ubiquitous. It is recognized that clinical depression carries potential to adversely affect recovery rates, the development of disability, and even mortality in cardiac patients (25). Assessing the presence of depressive symptoms associated with death perceptions may lead to interventions that could prevent progression into clinical depression. Little research has examined the presence of death depression in patients who have sustained AMI in Iran. Although Iran’s regional and cultural context may have a significant effect on people’s perception about death, (3) there is no standard tool for assessing death depression in these patients. Given this gap, it is essential to have a specialized tool that is able to accurately assess the presence and degree of death depression in patients who have experienced AMI. Health practitioners who are able to assess death depression in patients early may be able to target resources to prevent progression to clinical depression (26). Therefore, the present study aims at determining the psychometric properties of the DDS-R in Iranian patients with AMI.

## Materials and Methods

A psychometric analysis was conducted in 2016 following the collection of data from a convenience sample of 407 patients who had experienced AMI (September to December 2015). Sample size was determined based on the standard criteria identifying a minimum sample size for factor analysis, which is 5 to10 times more than the number of the instrument items (27). To ensure that the structure of the DDS-R was adequate, the minimum sample size for structural equation model (SEM) was estimated to be 387 according to an effect size of 0.1, statistical power level of 0.8, 4 latent variables, and 16 observed variables (28, 29).

The patients who were post AMI were recovering in the coronary care units (CCU) of 2 regional hospitals in Sari and Amol in Iran. The response rate of participants was 88.4%. The AMI diagnostic criteria were determined by a cardiologist and were related to ST segment changes in the electrocardiogram (ECG). ST elevation MI (STEMI) is an AMI characterized by ST elevation more than 0.2 mV in leads of V1 to V4 or over 0.1 mV in leads of I, II, III, aVL, aVF, V5, and V6. Non-ST elevation myocardial infarction (NSTEMI) is an AMI characterized by the existence of angina for more than 20 minutes along with an increase in a cardiac biochemical marker of myocardial necrosis (troponin or creatine kinase-MB), ST segment changes >1•0 mm but <2•0mm in V1 to V4, ST segment depression >1•0 mm at 80 ms, following the J point and inverted T and Pathological Q waves (duration _0•03s amplitude Q: R ratio _25%) (11).

A demographic questionnaire was used to collect information on the patients’ age, gender, educational, and socioeconomic status. The 21-item DDS-R was used to assess death depression. Initially, written permission was obtained from the DDS-R scale developer for study use. The World Health Organization protocol was used to translate the DDS-R into Persian (30). We employed the forward-backward translation technique to translate the scale from English into Persian. Accordingly, 2 English-Persian translators were invited to independently translate the DDS-R. An expert panel consisting of the study investigators and the 2 translators assessed and unified the 2 translations and produced a single Persian translation of DDS-R. Then, a Persian-English translator was asked to back-translate the Persian DDS-R into English. This English version of the DDS-R was sent to Dr. Templer, the developer of the questionnaire. He confirmed the correctness of the translations and the similarity of the recreated DDS-R with the original English version of the DDS-R. 

The DDS-R is scored on a 5-point Likert scale from 1 (completely disagree) to 5 (completely agree), ranging from 21 to 105, with lower scores showing lower levels of depressive symptoms regarding death (31). Face, content, and construct validity were considered. 

*1.* *Face validity Assessment*

The face validity of the Persian DDS-R was assessed both qualitatively and quantitatively.

*1.1. Qualitative Face Validity Assessment*

The qualitative face validity of the Persian DDS-R was assessed by inviting 10 cardiac patients to assess and comment on the appropriateness, difficulty, relevance, and ambiguity of the instrument items. The time needed for scale completion was also determined by this assessment. 

*1.2.* *Quantitative Face Validity Assessment*

The item impact technique was adopted to assess the quantitative face validity of the Persian translation of the DDS-R. The same 10 patients were asked to determine the importance of the individual items on a Likert-type scale from 1 (not important) to 5 (completely important). The individual item impact score was calculated by using the following formula: Importance Frequency (%). Using this formula, the frequency is equal to the number of patients who had obtained a score of 4 or 5 to the intended item, and importance was equal to scores 4 or 5 on the Likert scale. If the impact score of each item was greater than 1.5, the item was considered suitable and maintained in the scale (32, 33).

*2.* *Content Validity Assessment*

The content validity of the Persian DDS-R was also assessed both qualitatively and quantitatively as explained below.

*2.1.* *Qualitative Content Validity Assessment*

To assess qualitative content validity, the translated DDS-R was provided to 15 experts (9 nursing doctorates, 2 psychiatrists, 2 clinical psychologists, and 2 cardiologists). The experts were asked to assess and comment on wording, item allocation, and scaling of the items (34). Guided by this input, we subsequently revised the DDS-R.

*2.2.* *Quantitative Content Validity Assessment*

Content validity ratio (CVR) and content validity index (CVI) were assessed for the scale items. CVR reflects whether or not the items are essential. Accordingly, the same previously mentioned 15 experts were asked to rate how essential the DDS-R items were on a 3-point scale, the ratings are as follow: not essential: 1; useful but not essential: 2; and essential: 3 (35). The CVR of each item was calculated using the following formula: CVR = (NE – (N/2)) / (N/2). Using this formula, N and NE are respectively equal to the total number of experts and the number of experts who score the intended item as ‘essential’. According to Lawshe, when the number of panelists is 15, the minimum acceptable CVR is equal to 0.49 (36).

CVI can be calculated for each item of a scale (item -level; I-CVI) and for the overall scale (Scale-level; S-CVI). Accordingly, we asked the same 15 experts to rate the relevancy of the DDS-R items on a 4-point Likert scale from 1 to 4. The 4 points for rating the relevance of the items ranged from 1 (not relevant) to 4 (highly relevant). I-CVI of each item was calculated by dividing the number of experts who had rated that item as 3 or 4 by the total number of experts. Lynn et al. noted that when the number of experts is equal to 15, the items which acquire an I-CVI value of 0.79 or greater are considered appropriate (37). 

*3.* *Construct Validity Assessment*

**Figure 1:**
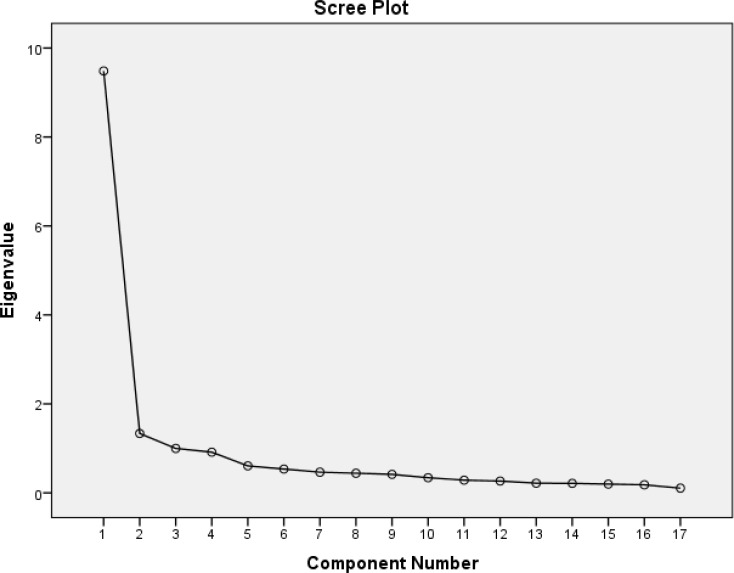
Scree Plot of Conducting an Exploratory Factor Analysis on the Persian Version of Death Depression Scale in Patients with Acute Myocardial Infarction

To examine DDS-R scale construct validity, we performed both exploratory factor analysis (EFA) and confirmatory factor analysis (CFA), and convergent validity and discriminant validity (38). We applied maximum likelihood (ML) with Promax rotational procedures by SPSS 22 (SPSS Inc., Chicago, IL, USA). The Kaiser–Meyer–Olkin (KMO) and Bartlett’s test of sphericity were used to check the appropriateness of the sample to conduct factor analysis. The number of factors extracted was based on eigenvalues and the Scree plot as shown in Figure 1. Eigenvalues greater than 1, which satisfied the Scree plot requirements of factor loadings greater than .5, were the criteria used to select factors (39-42).

Next, the results obtained from ML were confirmed by performing CFA with AMOS 21. Given the CFA output consisting of Chi-square (^2^) test, Chi-square/degree of freedom ratio (normalized chi-square CMIN/DF) < 3, Goodness-of-fit index (GFI) > 0.90, Comparative Fit Index (CFI) > 0.90, Incremental Fit Index (IFI) > 0.90, Normed Fit Index (NFI) > 0.90, and Root Mean Square Error of Approximation (RMSEA) < 0.08 were used for confirmatory factor analysis (43). 

Finally, convergent validity and discriminant validity of the factors were evaluated using average variance extracted (AVE), maximum shared squared variance (MSV), and average shared square variance (ASV). AVE of .50 or above indicates good convergent reliability. To establish discriminant validity, AVE should be greater than both MSV and ASV (44, 45).

*4.* *Reliability Assessment*

The internal consistency (reliability) of the Persian version of DDS-R was assessed using the coefficients of Cronbach’s alpha (α), Theta (θ), and McDonald Omega (Ω) (46). Values of .7 or greater show satisfactory internal consistency (45). Evaluation of Intra-class Correlation Coefficients (ICC) were used to establish the test–retest reliability of the DDS-R over a 2-week interval, using two-way mixed ICC for absolute agreement at the level of individual items (47). ICC was also calculated, and its results were interpreted as follow: 0 .0–0.2 as low; 0.21–0.40 as fair; 0.41–0.60 as moderate; 0.61–0.80 as substantial; and 0.81–1 as excellent (48). Next, construct reliability (CR) of the factors were assessed. CR greater than .7 indicates good reliability (45, 49). 

*Ethical Considerations*

The study was approved by the Ethics Committee of Mazandaran University of Medical Sciences, Sari, Iran (Ethics Code 410). Patients were informed about the study objectives and procedures. Moreover, they were ensured that participation was voluntary and it would not affect their medical management. Confidentiality of the participants’ information was assured by collecting data in private locations, assigning numbers rather than names to study documents, and avoiding the use of patient identifiers. Because more than half of the sample did not have formal education (see Table 1), the surveys were read to these participants by a trained research assistant to gain their responses. Informed consent was obtained from all participants.

## Results

Table 1 demonstrates the demographic and health information of the participants. Participants age ranged from 35 to 96 years and most of them were male (55%). The impact score, CVR, and I-CVI values of all 21 items of the Persian DDS-R were respectively greater than 1.5, 0.49, and 0.79. Therefore, none of the items were excluded in these steps of psychometric evaluation. Table 2 displays the results of the EFA using ML with Promax rotation on the Persian Version of DDS-R. Promax rotation allows factors to be correlated 30. ML extracted 4 factors consisting of a 6-item factor (eigenvalue = 3.932), a 4- item factor (eigenvalue = 2.649), a 4-item factor (eigenvalue = 2.228), and another 2-item factor (eigenvalue = 1.313). The analysis revealed 4 factors together accounting for 75.894% of the variance.

**Figure 2:**
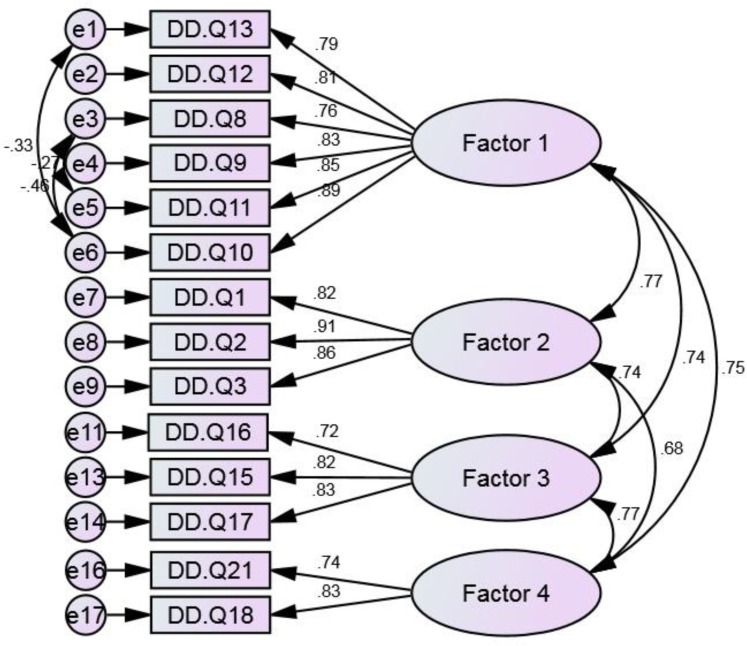
Confirmatory Factor Analysis of the the Persian Version of Death Depression Scale in Patients with Acute Myocardial Infarction.

Note: χ^2^(68) = 323.037, *p* < .05, χ^2^/df= 4.751, GFI = .903, CFI = .939, NFI = .924, IFI = .939, RMSEA (90% C.I.) = .096 (.086 - .107)

Next, using maximum likelihood CFA, we sought to confirm and validate the factor structure obtained from ML. As shown in Figure 2, the results indicated that the initial measurement model consisting of 4 extracted factors did not fit the data well [( ^2^ (98) = 583.646, *p* < .05,  ^2^/*df* = 5.956, GFI = .856, CFI = .902, NFI = .885, IFI = .902, RMSEA (90% C.I.) = .110 (.102 - .119)]. The final model was determined after removing items Q4 and Q20 from Factor 2 and 3, respectively, due to insufficient discriminant validity and by reviewing model modification indices for sources of model misfit. As shown in Figure 2, 3 pairs of item measurement errors of Factor 1 were allowed to freely co-vary to improve the model fit. Following the modification indices during DDS-R construction, there were correlations between the 10th and 13th items (between e1 and e6), the 8th and 10th items (between e3 and e6), and the 8th and 11th items (between e3 and e5). The revised model was found to be a good fit, as evidenced by goodness of fit indexes [( ^2^ (68) = 323.037, *p* < .05,  ^2^/*df* = 4.751, GFI = .903, CFI = .939, NFI = .924, IFI = .939, RMSEA (90% C.I.) = .096 (.086 - .107)], and significant factor loadings greater than .7 (z-value range 13.981 to 22.201). The significant χ2 was because of a relatively large sample size being used (45, 49). The internal consistency rate revealed good reliability and internal consistency for all factors. The average measure ICC was .955, with a 95% confidence interval from .948 to .961 (F = 23.67, *p*<.001). As reported in Table 3, the CR of all factors varied from 0.760 to 0.919, which indicates good reliability. Moreover, as AVE of factors exceeded .5 and construct reliability was greater than AVE, convergent validity was demonstrated. Furthermore, AVE was greater than both MSV and ASV, indicating discriminant validity.

**Table 1:** Demographic and Clinical Profile of the Participants with Acute Myocardial Infarction

**Characteristic**	**N (%)**	**Characteristic**	**N (%)**
Gender		CABG. Candidate	
Male	224(55)	Yes	127(31.2)
Female	183(45)	No	280(68.8)
Economic Situation		Smoking	
Weak	126(31)	Yes	166(40.8)
Average	168(41.3)	No	241(59.2)
Good	106(26)	Drug. Use	
Excellent	7(1.7)	Yes	55(13.5)
Education		No	352(86.5)
Illiterate	221(54.3)	Blood Pressure	
Diploma-BS	178(43.7)	Yes	220(54.1)
MCs and above	8(2.0)	No	187(45.9)
Alcohol		ACS	
Yes	77(18.9)	Unstable Angina	129(31.7)
No	330(81.1)	Segment elevation MI	213(52.3)
Family Heart Disease		Infraction without elevation	65(16)
Yes	297(73)	MI. Position	
No	110(27)	Anterior	43(10.6)
Daily Activity		lower	58(14.3)
Low	183(45)	lateral	44(10.8)
Intermediate	132(32.4)	anterior lateral	31(7.6)
High	92(22.6)	posterior	51(12.5)
Depression History		general	50(12.3)
Yes	165(40.5)	Missing	130(31.9)
No	242(59.5)		
CHF		**Characteristic**	**Mean(SD)**
Yes	74(18.2)	Age	63.72(16.373)
No	333(81.8)	Diastolic	89.544(13.210)
Diabetes		Systolic	160.959(18.154)
Yes	144(35.4)		
No	263(64.6)		

**Table 2:** Factor Analysis for the Persian Version of Death Depression Scale in Patients with Acute Myocardial Infarction

**Factors**	**Factor’s Name**	**Items**	**Loading**	**h** ^2^	**% of ** **Variance**	**Eigenvalues**
1	**Fear of death**	Q13: It’s hard to concentrate when death is on my mind	.908	.741	56.193	3.932
		Q12: The thought of death saps my energy	.871	.808		
		Q8: The thought of death makes it difficult to experience pleasure	.870	.670		
		Q9: When I think about death, I lose interest in activities of life	.782	.744		
		Q11: When death is on my mind, my body seems to lose energy and slow down	.749	.740		
		Q10: I lose interest in caring for myself when I think about death	.650	.746		
2	**Sad farewell**	Q1: When I think about death, I feel empty	.954	.818	8.202	2.649
		Q2: Thinking about death makes me tearful	.868	.829		
		Q3: Dying must always be an unhappy process	.749	.777		
		Q4: Nothing saddens me more than knowing friends and relatives will eventually die	.652	.758		
3	**Hopelessness**	Q16: Why try in life if you are only going to die	.966	.811	6.177	2.228
		Q20: I am terribly upset by the shortness of life	.709	.715		
		Q15: Death makes me feel discouraged about the future	.632	.717		
		Q17: Death makes me feel hopeless	.627	.739		
4	**Fear of leaving ** **loved ones**	Q21: I dread to think of the death of friends and loved one	.881	.798	5.322	1.313
		Q18: Wakes and funerals are depressing	.716	.733		

Abbreviation: h2: Communalities

**Table 3:** Construct Validity and Reliability of the Factors of the Persian Version of Death Depression Scale in Patients with Acute Myocardial Infarction (Fornell Larcker Criterion Table)

	**α**	**θ**	**Ω**	**CR**	**AVE**	**MSV**	**ASV**	**Factor 1**	**Factor 2**	**Factor 3**	**Factor 4**
**Factor 1**	.919	.906	.918	.919	.655	.624	.587	.809			
**Factor 2**	.895	.835	.893	.899	.748	.624	.545	.790	.865		
**Factor 3**	.837	.741	.836	.835	.629	.588	.564	.742	.744	.793	
**Factor 4**	.754	.476	.921	.760	.614	.588	.544	.766	.676	.767	.783

Abbreviations: **α**: Cronbach’s alpha coefficients, **θ**: Theta Coefficient, **Ω:** McDonald Omega Coefficient, **CR**: Construct Reliability

**AVE**: Average Variance Extracted, **MSV**: Maximum Shared Squared Variance, **ASV**: Average Shared Square Variance

## Discussion

The results revealed that DDS-R has 4 dimensions that include items reflecting death concern, sad farewell, sense of despair, and leaving loved ones, respectively. The 4 extracted factors indicated 75.89% of the variance. According to a study by Rajabi et al., who recruited Iranian nurses, 3 factors of the death depression scale (i.e., other death, death sadness, anergia, and vacuum) were extracted, which showed a total of 65.64% of variance (50). A similar study on student found 4 factors (i.e., death despair, death failure, death loneliness, and death acceptance), 

indicating 49.71 of the variance (51). Hair, Black, Babin, and Anderson suggest that in psychological sciences studies the variance was 50% to 60% and factor extraction was appropriate (45). In another study conducted by Tomás-Sábado and Gómez-Benito assessing the validity of a Spanish version of the DDS-R, 4 factors (death sadness, death finality, meaninglessness of life and feeling of loss), with eigenvalues higher than 1 were identified (21). In the present study, the first extracted factor was death concern, which may reflect worries and anxiety about death (2). 

Given the recent AMI, fears associated with mortality are a significant threat to wellbeing and may contribute to ruminating concern (52). Ongoing unresolved death concerns could later become related to the development of depressive symptomatology (15).

The second factor was sad farewell. The set of items associated with this factor related to the impending perceptions of shortened life, negative affect associated with a sense that life is over and a limited opportunity to let go, and leave loved ones. If the patients were the family breadwinner, the perception of leaving family members could be particularly depressing (21).

The third factor indicated despair surrounding cognitions about death. Thoughts, beliefs, and attitudes towards death may fluctuate over time and may differ depending on lifestyle circumstances, cultures, and societies (1). Despair about death may surface secondary to cognitions originating from personal and social factors, loss of hope, and heightened concerns about the nature of death, and prescribed meanings (53). This factor has also been reported in other studies (21, 51).

The fourth factor relating to leaving loved ones is similarly related to negative cognitions and affect associated with the reality of death. Chan et al., state that a central concern for most people is accepting the loss of a tangible relationship with loved ones (54). Patients may become demoralized when considering that these temporal relationships are coming to an end. Such depressive cognitions may also be associated with death anxiety (55). This factor was similar with the other studies (21, 51).

In the current study, the fitness of the DDS-R factor structure was assessed after removing outliers, evaluating common weak indicators of model fitness, and also assessing the natural distribution of data via CFA. Results determined that model fitness indicators were appropriate, and factor loadings were over 0.5, identifying the minimum acceptable rate of factor loading. Thus, observed indicators were confirmed via CFA and all fitness indicators had a suitable standard level. To the best of our knowledge, other related studies evaluated EFA only (50, 56) with CFA evaluation a strength of the present study.

According to the final DDS-R model, there were correlations between the measurement errors of items 10th and 13th (e1, e6), 10th and 8th (e3, e6), and 11th and 8th (e3, e5). Munro (2005) stated that correlated measurement error occurs when variables have not been identified clearly or have not been measured directly, so that it can affect the responses to certain items (57). Hidden variables can be problems associated with actual scores of measurement error structures (58). Measurement errors can be created by the impact of measuring effectiveness with self-report questionnaires. Measurement errors can also result from the presence of similar words or phrases in items used to construct questionnaires (59).

DDS-R items in the final model had an appropriate structural convergent and divergent validity. Hare states that there is a convergent validity when the intended structural items are close to each other and share variance. Divergent validity is determined when intended structural items or the hidden extracted factors are completely separate from each other (45). In other words, there is no suitable convergent validity when the hidden factors are not well- explained by the extracted items and the items have no sufficient correlation with each other (44).

The reliability of DDS-R was found to be highly suitable in this sample of Iranian patients who had sustained an AMI. The ratings of coefficients of internal consistency suggests that the reliability of the questionnaire was appropriate and that there was a correlation between items, indicating that the questionnaire’s items are measuring similar concepts and no conceptual dispersion is seen. Moreover, internal consistency using Theta and McDonald Omega were acceptable. Templer, et al. reported the reliability of the original version of the questionnaire, with a Cronbach’s alpha coefficient of 0.92. Templer stated that the significant correlations between this tool and other depression and anxiety scales were reliable (31). The evaluation of the DDS-R’s reliability among Spanish students showed Cronbach’s alpha coefficients of 0.90 and test re-test correlation of 0.87 within 4 weeks (56). Rajabi et al. and Aghazadeh et al. reported the reliability of the questionnaire calculated by Cronbach’s alpha coefficients and test- retest method as 0.93 and 0.76, respectively (50, 51). In another study, the reliability of 0.83 (with Cronbach’s alpha) and 0.87 (with test- retest) was reported (21).

In the current study, CR was in its highest level. Indeed, CR or factor consistency is a kind of substitution for Cronbach’s alpha coefficients in the SEM (60). One of the important features of estimating CR rather than Cronbach’s alpha coefficients is that it is not affected by the number of scale items and the obtained structure is dependent on the actual factor loading of each items on latent variables (60). CR is considered to be more accurate than Cronbach’s Alpha. Few previous studies have calculated the DDS-R CR rate.

## Limitations

The forward-backward translation method was performed at a high standard in this study, and the original author of the scale confirmed the accuracy of the translation. However, there is always a potential difficulty in using scales that were originally designed for different populations. Cultural differences and language nuances may not be translatable in such questionnaires. Instrument users would be advised to be cognizant of such potential issues. Further, participants who did not have formal education background had the questionnaires read to them which could potentially bias their willingness to self-report. 

## Conclusion

The present findings suggest that the Persian version of the DDS-R has a 4-factor structure and acceptable validity and reliability in a sample of Iranian patients with post AMI status. This study demonstrates that a significant percentage of each DDS-R items’ variance is explainable in Iran’s cultural context. With respect to the importance of mental health and the prevention of negative psychiatric sequelae among patients with AMI, the existence of the DDS-R could be useful in accurate measurement of death depression.
